# Farmers’ perception on soil erosion in Ghana: Implication for developing sustainable soil management strategy

**DOI:** 10.1371/journal.pone.0242444

**Published:** 2021-03-02

**Authors:** Gebreyesus Brhane Tesfahunegn, Elias T. Ayuk, S. G. K. Adiku

**Affiliations:** 1 College of Agriculture, Aksum University-Shire Campus, Shire, Ethiopia; 2 United Nations University Institute for Natural Resources in Africa (UNU-INRA), University of Ghana, Campus Legon, Accra, Ghana; 3 Department of Soil Science, University of Ghana, Campus Legon, Accra, Ghana; International Institute of Tropical Agriculture, NIGERIA

## Abstract

Farmers’ perception on soil erosion has not adequately reported in the conditions of Ghana though its causes and effects are time and site-specific. The objective of this study was to assess farmers’ perception on soil erosion and implication for developing soil management strategy in the Eastern and Northern Regions of Ghana. A total of 130 household head farmers were interviewed and complemented with field observation and group. Data was analyzed using descriptive, chi-square test, T-test and binary logistic regression. The results show that there was significant variation in socioeconomic, farm and institutional attributes among the farmers`in the study regions. In the Eastern and Northern Regions, significantly higher proportions of the farmers (95.7% and 86.7%, respectively) perceived soil erosion as serious problem. Significantly higher proportions of the respondents (80%) perceived severe erosion problem at homestead land in the Eastern Region whereas severe erosion in the Northern Region was more noticed at distance farmlands (85.0%). In the two regions, the major causes of severe erosion as perceived by most farmers were over-cultivation, deforestation and heavy rainfall events. In the Eastern and Northern Regions, 58.6% and 75.0% of the farmers perceived, respectively, that soil erosion severity has been increased since the past 10-years. Perceptions of most frequently noted indicators of soil erosion were declined productivity, shallow soil depth, presence of rills, sheet erosion, soil loss from farmland, and change in soil color. Results of the binary logistic regression indicate that there is heterogeneity in the factors accounting for the perception of soil fertility. In developing promising soil management strategy in the study area, attention must be given to key socioeconomic, biophysical, farm and institutional factors.

## Introduction

Soil degradation due to erosion is a serious global problem in general and in developing countries in particular as the majority of the people heavily depends on improper use of natural resources. Globally, agriculture is the main driver for about 80% of the existing degradation due to erosion [1–3]. According to Nkonya et al. [3], degraded land covers about 30% of the total global land area and about three billion people are resided in such degraded lands. From the annually degraded soil at global level, more than 40% of the severely degraded soil is found in Africa. The largest share (22%) of the total global cost of land degradation (300 billion US Dollar) is estimated from Sub-Saharan Africa (SSA) countries as this region has experienced severe land degradation over the last many decades [2–4].

Soil erosion is one of the most important forms of soil degradation that threatens sustainable agricultural production in Ghana. It is the major constraint for achieving the desired growth level of agricultural production in the country [5–8]. For countries such as Ghana whose economy depend largely on the agricultural sector, agricultural productivity decreasing by erosion leads to poor socio-economic development [3, 5–7, 9, 10]. The most severely erosion affected areas in Ghana are found in the Northern Savanna Regions, where large mass of land is destroyed by water erosion that leads to poor soil fertility, reduction in soil depth, soil structure degradation, formation of rills and gullies and siltation of rivers and reservoirs [5, 8, 10, 11]. For instance, in the Savannah Zone of Ghana land area of 35,172 km^2^ is affected by slight-to-moderate sheet erosion, 27,306 km^2^ by moderate-to-very severe sheet and gully erosion and 33,494 km^2^ by moderate-to-very severe gully erosion [5, 10]. The causes for such severe erosion in Ghana are reported as poor agricultural practices, mining, infrastructure and urban expansion, firewood and charcoal production, illegal logging, bush fire and overgrazing [3, 12, 13].

The severity of erosion indicates that Ghana’s natural resources upon which the country’s economic activity and the population’s livelihood largely depend on are being depleted at an alarming rate. For example, more than 50% of the original forest area has been converted to agricultural land. Under such condition, however, crop yields are gradually stagnated as soil productivity has declined immediately due to severe soil erosion and nutrient depletion [14–16]. Despite of the severe impact of soil degradation on ecology and human welfare and development, investments in sustainable soil and land management are low in countries such as Ghana [3, 15, 16]. This implies that there is a need to have sufficient research outputs that support to develop appropriate action against soil degradation due to erosion in the Ghana conditions.

Several other researchers have reported that the land of Ghana is under threat of desertification, especially, the Upper East, Upper West and Northern Regions of the country [5, 10, 11, 17–19]. Soil degradation due to erosion in the Northern Region of Ghana has rendered large tracts of croplands which were once fertile some years ago but currently being changed to unproductive land. As a result, natural water bodies are drying up due to increasing sediment deposition into water courses [10, 11, 16, 19]. Rapid soil degradation has also been reported since the last two decades in the Eastern Regions of Ghana [5, 10, 20, 21].

Despite of the above reports about soil degradation, the nature and extent of farmers’ knowledge and perception on soil degradation due to water erosion has not been sufficiently understood in the conditions of Ghana as the causes and effects of degradation are time and site-specific [16, 22–24]. Many technological and institutional innovations that can reduce degradation have been developed and introduced; and yet, such innovations do not generally appear to be successful to reduce the severity of degradation [16, 25–28]. Some of the main reasons for such unsuccessful interventions against soil degradation could be lack of fit between proposed techniques and local farming systems, poor platform to consider local knowledge, limited available inputs, poor market channel, inappropriate land tenure and poor participation of local people in designing, implementing, monitoring and evaluation of technologies [16, 29, 30].

In the existing literature, there is variability in approaches used to assess soil degradation due to erosion as some are based on descriptive statistics and others are based on combinations of both qualitative and quantitative research, indicating the need to assess properly using site-specific approaches. The implication is that soil degradation can be assessed through a number of scientific methodologies. These included: satellite remote sensing and geographic information system (GIS) that analyze changes in land use and land cover effects on erosion [31–34], ecological assessment models [35–38], and measurement of soil properties [36, 39, 40]. Previous reports have also appreciated that the two approaches of scientific and local knowledge can integrate to complement to each other to provide holistic assessment of soil erosion [38, 39, 41]. Others have reported that understanding of local knowledge (farmers) about erosion problem can support to be able to fully realize their capacity to monitor and respond more quickly to changes and challenges than the scientific techniques [35, 38, 39, 42]. Hence, this study has attempted to answer the research question ‘How do farmers perceive soil erosion problem, causes and effects, indicators and the determinant factors in the context of Ghana?’

Analyzing erosion problem based on farmers’ perception can provide quick and practical information that is essential for sustainable soil management and land use planning [5, 16, 39, 41]. The determinant factors for farmers’ perception on erosion can vary by resources availability, agro-ecology and socioeconomic conditions of the farming community. However, research about farmers’ perception on soil degradation by erosion, causes, indicators, and determinant factors that help to develop management options, have been given little attention in many developing countries including Ghana [16, 38, 39, 41].

Existing evidences have shown that local people such as farmers have significant knowledge on soil degradation indicators and its economical, social and environment consequences. Such experiences have acquired and also tested practically by the farmers for many generations living close to their land [5, 39, 43]. Understanding farmers’ knowledge on soil degradation due to erosion is crucial for successful introduction of development efforts [39, 41, 43]. To design more appropriate landscape scale soil management practices that can disseminate widely to farmers, there is a need to understand farmers’ perception on soil erosion problem [38, 39, 41, 44]. However, there are insufficient scientific reports on how the local people in general and the farmers in particular understood soil degradation due to erosion in the conditions of the Eastern and Northern Regions of Ghana. The objective of this study was to examine farmers’ perception on soil degradation due to soil erosion in the Eastern and Northern Regions of Ghana and its implication for developing sustainable soil management strategy.

## Materials and methods

### Study area description

**Fig 1:**
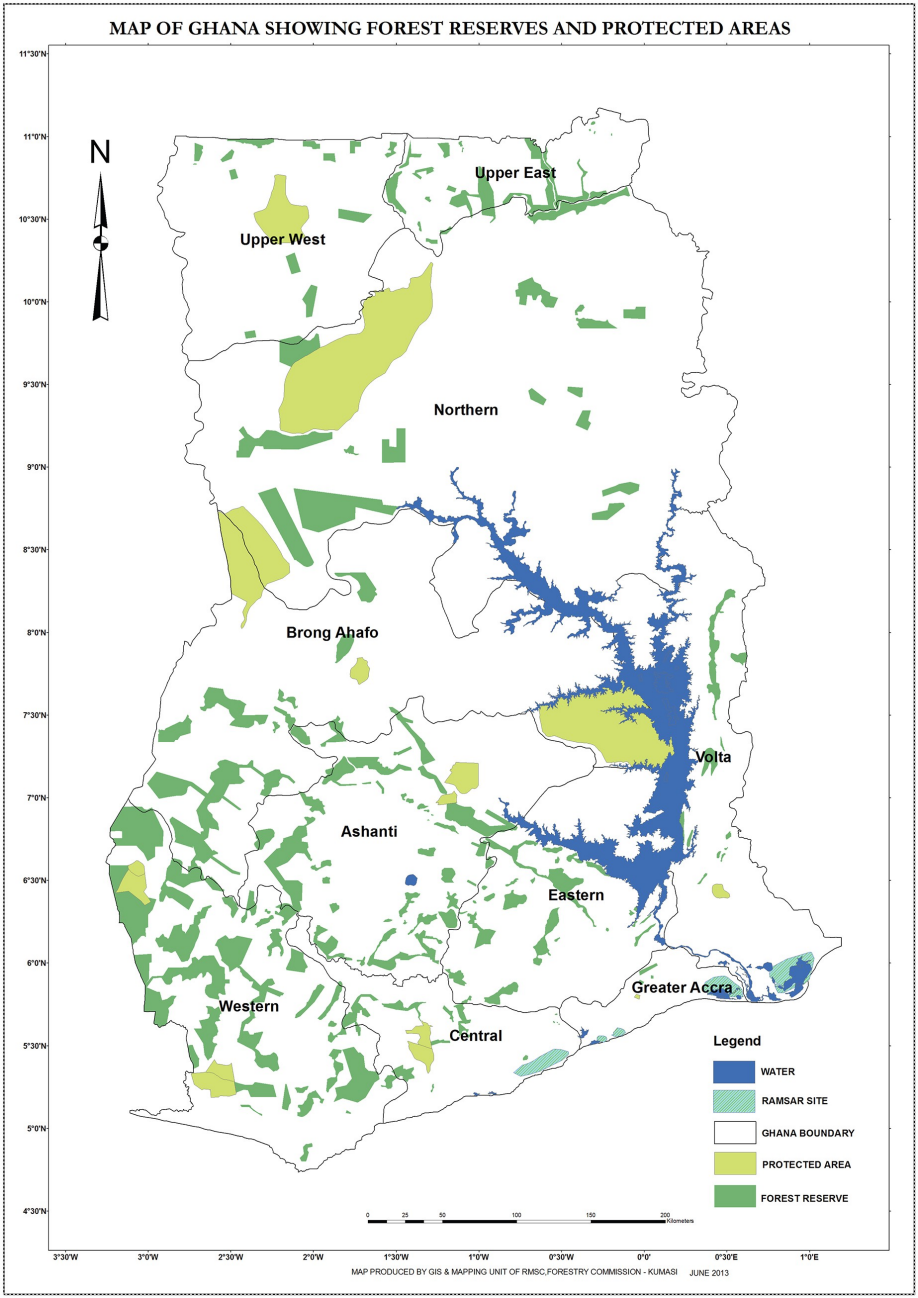
Map of Ghana with all the regions including the Eastern and Northern. (Source: Ghana Ministry of Lands and Natural Resources [45]).

This study was executed from Jan 2017 to July 2017 in the Eastern and Northern Regions of Ghana (Fig 1). The Eastern Region lies between latitudes of 60^o^ North and 70^o^ North, which covers an area of 8.1% of the Ghana’s total landform. This Region falls almost within the two main vegetation zones, namely, the Tropical Forest Zone (60%), and the Guinea Savanna Zone (40% [45]. The Eastern Region was selected as a study area because there are limited studies about the problem of soil degradation due to soil erosion even though soil erosion has been rapidly increased from time to time. The Eastern Region also captures variability in soil fertility, altitude, cropping and livestock systems, and forest and other land use/ land cover types that influence farmers’ perception on soil erosion [20, 21]. One village, namely, *Brepaw Kpeti* was purposively selected from the Upper Manya district in the Eastern Region of Ghana. This village is located at N 06^o^24’39” and W 000^o^06’26” and has average altitude of 450 meter above sea level. In this village, annual rainfall and temperature ranges between 900 and 1500 mm, and 26 and 32^°^C, respectively (District Bureau of Agriculture, Unpublished Report in 2017).

The Northern Region of Ghana which is the other study area is much drier than the Eastern parts of Ghana, due to its proximity to the Sahel and the Sahara belt. *Nyankpala* was the study village which was purposively selected from the Tolon district in the Northern Region as this represents erosion severe sites and also its accessibility for frequently field works. In *Nyankpala* village, annual rainfall and temperatures ranges from 750 to 1050 mm, and 14 to 40°C, respectively. The mean elevation of the study village is 183 meter above sea level. This village is located at N09^o^24’ and W000^o^60’. The predominant vegetation consists of savanna grassland and clusters of drought-resistant trees such as *acacias*. In this region, more than 75% of the economically active population are involved in agriculture [45].

### Sampling technique and sample size

In this study, the village *Brepaw Kpeti* from the Eastern Region and *Nyankpala* village from the Northern Region were purposively selected. The rational used during the villages selection included: access to transportation for frequent field visits, and presence of high indicators of land degradation (biodiversity loss, soil degradation). The selection of the villages was done in consultation with two extension staffs from the district Bureau of Agriculture and two farmers’ contact persons in each of the study villages. In addition, field reconnaissance survey was conducted to check the suitability of the selected villages and so verified the information acquired from the extension staffs and farmers contact persons. From each of the study villages total population, the sample of farmers’ household heads were selected using systematic random sampling at 95% confidence level or 5% margin of error. A total of 130 household head farmers, i.e., 70 farmers from the village *Brepaw Kpeti* in the Eastern Region and 60 farmers from the village *Nyankpala* in the Northern Region of Ghana were interviewed. Such sample size was determined using Asmamaw [46] formula from a total population of 85 farmer household heads in *Brepaw Kpeti* village; and 71 household heads in the *Nyankpala* village.

### Data sources and types

In this study, two sources of data, which were primary and secondary sources, were used. The primarily data were collected using field observation, group discussion and questionnaire interview. Both qualitative and quantitative data types were collected. Available secondary data such as climate/weather from meteorology agency, soil, land use types, agricultural practices, demography, were collected from published and unpublished sources such as reports. The detail procedure for the primary data collection is shown below.

#### Field observation and group discussions

Different indicators of erosion, sedimentation, runoff, and soil and water conservation practices were observed at field level. Discussion was held about erosion severity and the corresponding indicators observed at filed level with a team composed of four members. For the field observation, a team consisted of one researcher, one extension staff, and two farmers contact persons was established. In the group discussions, two village leaders, two extension staffs, three farmers and one district agricultural official were participated. The six (6) participants in the group discussions except the farmers were purposively selected as they knew the village condition from the perspective of erosion and resources management. The farmers participated in the group discussions were selected randomly from the villagers. The farmers who participated in the group discussions were not involved in the semi-structured interview. Three discussion events were conducted in order to get clear information about the severity of the erosion and its causes and effects in the study villages. Such discussions were used as a means to crosscheck data collected about farmers’ perception on soil degradation due to erosion using the formal interviews.

#### Semi-structured questionnaire interview

Semi-structured questionnaire was developed and pre-tested using randomly selected 12 farmers from each of the two study villages. Some minor revision was done on the flow of the questions in the questionnaire based on the feed-back obtained from the pre-tested result. The questionnaire had two main sections: (1) household socioeconomic, farm and institutional attributes, and (2) farmers perceptions on erosion problem, causes and effects, and indicators of soil erosion. The questionnaire interviews were administered face to face by employed two enumerators for each of the study villages, with close supervision from the first author. Orientations were given to all enumerators on how to translate the questionnaire from English into local language while interviewed the farmers’ household heads. In the Eastern Region, the farmers spoke the local language *Krobo* whereas in the Northern Region they spoke *Dagban*. Religion of all respondents in the Eastern Region was Christian and that of the Northern Region was Muslims.

### Data analysis

SPSS version 20 software was used to analyse the data collected from the two study villages. Specifically, descriptive, non-parametric (e.g., chi-square test) and T-test and econometric analyses were used at the probability level (P) ≤ 0.05. The chi-square test was used to assess the statistical significance between the proportions of the respondents’ replied in favour of a certain question. Binary logistic regression was used to analyze the causal-effects of the explanatory variables on the dependent variable (farmers’ perceived on soil erosion as problem) at P ≤ 0.05. The logistic model was selected over the others such as probit because it is more descriptive nature and interpretable for data related to perception on erosion [38, 47]. The dependent variable is farmers’ perceived that erosion is a problem. If a farmer perceived soil erosion as a problem, it is denoted as 1 (yes), 0 otherwise. Where 1 indicates the presence of perception and 0 indicates the absence of perception on the attribute. The independent variables included in this study were: age, gender, education, family size, literacy ratio (illiterate/literate), dependency ratio (dependents/productive), total number of livestock, farm size, land tenure, farmland ownership, farming experience, experiences on soil management practices, off-farm activities, income from agriculture, access to extension services such as training, access to information (media, radio), access to credit, slope of farm land and arable land distance from home and main road. The hypothesised relationships of such variables with the dependent variable were determined based on the existing literature and the researchers’ judgment (Table 1).

**Table 1:** Description of variables used in the analysis of farmers’ perception on soil erosion.

Variable	Ho	Explanation of effects
Age of the HH (year)	+	It is hypothesized that older farmer is experienced and thus have a higher likelihood of perceiving soil erosion degradation than younger farmers [48].
Gender of the HH (1 = male, 0 = female)	+	Male headed HH have better access to experience sharing events than female and thus influences positively perception on soil degradation [38, 48].
Education (1 literate, 0 = Illiterate)	+	Education increases ability of HH to practices options of soil management and thus hypothesized to influence positively perception on soil degradation [49, 50].
Literacy ratio (dimensionless)	-	The higher in literacy ratio implies more illiterates and this affects negatively farmers’ perception on soil degradation due to erosion [38].
Family size (number)	+	A bigger family size of HH who involved on farming can share soil degradation experiences and thus affects positively the perception on soil erosion [38, 49].
Dependency ratio (dimensionless number)	-	The higher dependency ratio implies more dependants and this affects negatively the perception on soil degradation due to erosion [38].
Farming experiences (year)	+	The higher the number of farming experiences the better perception on soil erosion problems.
Total livestock owned (number)	+	A higher number of livestock owned by HH experienced effects on erosion and thus influences positively farmer perception on soil erosion. It is a continuous variable [48].
Farmland size (ha)	+	A farmer having larger farmland size could have a chance to see effects of erosion variation and thus hypothesized to influence positively perception on soil degradation [38].
Slope of farmland (1 = steep, 0 = flat)	+	Steep field slope increases the effects of erosion and it is hypothesized to be positively associated with farmer perception on soil degradation [38, 49].
Land ownership (1 = own, 0 = rent)	+	Farmers who owned land for many years can better perceive erosion on their land and thus affects positively their perception on erosion [48].
Land tenured (1 = yes, 0 = no)	+	A farmer with tenured land is hypothesized to influence positively perception on soil degradation as farmer interventions considering land tenured [51, 52].
Farm land distance from home/main road (km)	-	As farmland distance increases from home/main road this influences negatively for farmer perception on soil erosion, there is less opportunity to visit it frequently [49].
Total agricultural income (number)	+	This indicates that farmers who earned a higher income from agriculture improve their perception positively on soil degradation as this helps to practice suitable technologies [38].
Off-farm income (1 = yes, 0 = no)	-	A farmer headed HH who mainly involves on off-farm activities may not perceive soil degradation and thus hypothesized to influence negatively their perception [52, 53].
Access to extension (1 = yes, 0 = no)	+	Access to extension service by farmer HH is fundamental to receiving information and technology and is hypothesized to positively influence perception on erosion [38, 52, 53].
Access to credit/ association (1 = yes, 0 = no)	+	Access to credit/ association to support agricultural inputs and watershed management, is more likely to influence positively understanding on soil degradation [38, 54].

Note: H_0_ indicates a hypothesized effect of an explanatory variable on the dependent variable which is farmers perceived soil erosion as problem (1 = yeas, 0 = no); + is positive and–is negative effect. HH is household head.

Multicollinearity among the explanatory variables was tested separately for continuous and dummy/discrete variables before the analysis conducted using the binary logistic regression. Variance Inflation Factor (VIF) was used to detect multi-collinearity among the continuous independent variables whereas contingency coefficient (CC) was used for the dummy or discrete variables. As a rule of thumb, if the VIF of the association among the variables exceeds 10; there is a strong multi-colinearity problem and should be excluded the non-significant variables from the analysis [55–57]. The CC values vary between 0 and 1; in which zero indicates there is no association between variables while values close to 1 indicates high degree of association between variables. The association is said to be high when the value of CC exceeds 0.75 [55, 56]. In this study, the analysis results showed that the values of VIF and CC among the independent variables were within the lower level of association (data not shown) which indicates that there is no serious problem of multi-collinearity effect among most of the explanatory variables. Strong multi-collinearity was only detected between the variables ‘education and literacy ratio’, and ‘total family size and dependency ratio’, in which literacy ratio and dependency ratio were excluded from the analysis as these were non-significantly influenced the dependent variable.

## Ethics statement

Ethical approval was obtained to conduct this study from the Research Director Research Ethics Review Committee of United Nations University Institute for Natural Resources in Africa (UNU-INRA), University of Ghana, Campus Legon, Accra, Ghana. Before executed the interview, brief introduction was given about the purpose of the study for the District Office of Agriculture staffs. The same briefing was given for the farmers in the presence of the extension staffs. The farmers were also requested orally for their consent to involve in the study. Full right was given to the study participants to refuse and withdraw their participation at any time. Confidentiality of respondents was preserved by the researcher and enumerators during the questionnaire interview. It was also noted that this research has no any other activity that directly influences on human being life as data were collected using an interview approach. It is a normal process to contact respondents for such data collection after introducing about the objective of the study and showing the approved proposal for the District Office of Agriculture extension staff. The extension staff is a focal person in the Office of Agriculture who facilitated directly our communication with the farmers (respondents) in the study villages related to this research.

## Results and discussion

### Qualitative farmers socioeconomic, farm and institutional attributes

The qualitative result of farmers’ socioeconomic, farm and institutional attributes from the Eastern and Northern Regions of Ghana study villages are shown in Tables 2 and 3, respectively. Except farmland slope and access to association in the Eastern Region study village, and off-farm activity and access to extension services in the Northern Region, the chi-square test results showed significant differences among the proportions of the respondents’ who replied with respect to a specific attribute. For example, in the Eastern Region of Ghana, male and female farmers household heads involved in the interview were 91.4 and 8.6%, respectively; indicates that the proportion of male respondents were significantly higher than that of the female respondents. In this region study village, about 78.6% of the respondents were married and the rest were either divorced or widowed, indicating that the proportion of married household heads were significantly higher than the other marital status.

**Table 2:** Qualitative results of respondents’ socioeconomic, farm and institutional attributes tested using chi-square test in the *Brepaw Kpeti* village, Eastern Region of Ghana (n = 70).

Attribute	Value	Attribute	Value
**Gender**	**	**Main occupation**	**
Male	64 (91.4)	Agriculture	63 (90.0)
Female	6 (8.6)	Daily laborer	2 (2.9)
**Martial status**	**	Petty trading	5 (7.1)
Married (live together)	55 (78.6)	**Off-farm activity**	**
Divorced	4(5.7)	Yes	65 (92.9)
Widowed	11 (15.7)	No	5 (7.1)
**Education (illiterate vs. literate)**	**	**Access to extension service**	**
Illiterate	18 (25.7)	Yes	42 (60.0)
Literate as informal education	3 (4.3)	No	28 (40.0)
Primarily education (up to Junior high school)	28 (40.0)	**Access to credit**	**
Secondary education (senior high school)	19 (21.1)	Yes	12 (17.1)
College	2 (2.9)	No	58 (82.9)
**Farmland slope**	Ns	**Access to radio**	*
Flat to gentle	31(44.3)	Yes	45 (64.3)
Gentle to steep	39 (55.7)	No	25 (35.7)
**Land tenured**	**	**Access to local association**	**ns**
Yes	64 (91.4)	Yes	41 (58.6)
No	6 (8.6)	No	29 (41.4)
**Rented out land**	**	**Own land**	**
Yes	6 (8.6)	Yes	65 (92.9)
No	64 (91.4)	No	5 (7.1)

Values in parentheses are percentages

** and *, chi-square test significant at P ≤ 0.01 and P ≤ 0.05, respectively; ns, non-significant at P > 0.05.

(Source: Own Survey Data, 2017).

**Table 3:** Qualitative results of respondents’ socioeconomic, farm and institutional attributes tested using chi-square test in the *Nyankpala* village, Northern Region of Ghana (n = 60).

Attribute	Value	Attribute	Value
**Gender**	**	**Main occupation**	**
Male	57 (95.0)	Agriculture	60 (100)
Female	3(5.0)	Daily labor	5 (8.30)
**Martial status**	**	Petty trading	12 (20.0)
Married (live together)	55(91.7)	Other sources such as driver	15 (25.0)
Divorced	3(5.0)	**Off-farm activity**	**ns**
Widowed	2 (3.3)	Yes	34 (56.7)
Single	0 (0)	No	26 (43.3)
**Education (illiterate vs. literate)**	**	**Access to extension service**	**ns**
Illiterate	46 (76.7)	Yes	28 (46.7)
Informal education	8 (13.3)	No	32 (53.3)
Primarily education (up to Junior high school)	4 (6.7)	**Access to credit**	**
Secondary education (senior high school)	0 (0)	Yes	2 (3.3)
College	2 (3.3)	No	58 (96.7)
**Farmland slope**	*	**Access to radio**	*
Flat to gentle	40 (66.7)	Yes	45 (64.3)
Gentle to steep	20 (33.3)	No	25 (35.7)
**Land tenured**	**	**Access to local association**	**
Yes	59 (98.3)	Yes	9 (15.0)
No	1 (1.7)	No	51 (85.0)

Values in parentheses are percentages

** and *, significant at P ≤ 0.01 and P ≤ 0.05, respectively; ns, non-significant at P > 0.05. (Source: Own Survey Data, 2017).

In the study village from the Eastern Region, the proportions of farmer respondents’ education status indicated that about 25.7% of the respondents were illiterate and the remaining were literates, indicating that education can influence positively farmers’ perception on soil erosion. The majority of the respondents (55.6%) in the study village from the Eastern Region were possessed flat to gentle farmland. Significantly higher proportions of the respondents (91.4%) had a feeling of land secured and also did not rent out their land. In the study village, 90% of the interviewed farmers’ main occupation was agriculture (Table 2). About 92.9% of the respondents in the Eastern Region study village were involved on off-farm activities even though this is not their main economic occupation. In this region, the respondents who accessed to services such as extension services, credit, media (radio), and membership to association were 60.0, 17.1, 64.3 and 58.6%, respectively.

In the study village from the Northern Region of Ghana, the proportions of male respondents (95%) were significantly higher than that of the female household heads (5.0%). In this region, significantly higher proportions of the respondents (91.7%) were married. In the Northern Region, the proportions of respondents in the different education status varied significantly, with the higher proportions of the respondents were illiterate (76.7%) (Table 3). In this region study village, the majority of the respondents (66.7%) possessed flat to gentle slope farmland. Significantly higher proportions of the respondents (98.3%) of the study village in the Northern Region felt land secured or tenured. In addition, all of the respondents in the study village from this region were engaged on agriculture as their main occupation. However, the majority of the farmers did not have access to extension services (53.3%) and credit (96.7%) services in the study village from the Northern Region. About 64.3% of the household heads had accessible to radio, but 85% of them were not member of development association in their locality (Table 3).

In agreement to the present result of significant variation in qualitative attributes among the farmers in the two regions, previous reports have reported that farmers socioeconomic attributes (e.g., education, gender, marital status, agricultural occupation); farm characteristics such as slope of farmland, land tenure; and institutional factors (e.g., access to extension services, credit, association) could influence differently farmers perception on soil erosion. For example, when the farmers’ education level increases, this influences positively to perceive on soil degradation [38, 49, 50, 58]. Several researchers have also reported that differences in socioeconomic characteristics of local farmers could influence significantly their demand for extension services and thereby on their perception on land degradation [38, 49, 50, 58, 59].

### Quantitative farmers socioeconomic, farm and institutional attributes

In the Eastern Region of Ghana, the respondents’ age, total family size, and farming experience varied significantly from 31 to 79 years, 3 to 16, and 13 to 55 years, respectively. In this region, a household farmer owned a total livestock number between 0 and 64. Almost all of the respondents in the study village of the Eastern Region were dependent on rain-fed agriculture (Table 4). A household head farmer total farmland size that included owned land, rented and inherited land, varied significantly from 0.61 to 15.4 ha, with a mean of 3.25 ha. However, the farmland size owned by a household head farmer ranged between 0.0 and 6.27 ha, with a mean farmland size of 2.13 ha. This mean farmland size owned by a farmer is slightly lower than the report by Peprah et al. [16] who reported that more than 70% of the farmers cultivated upto 3 ha of land in Ghana.

**Table 4:** Quantitative results of respondents’ socioeconomic, farm and institutional attributes of the study village from the Eastern Region of Ghana (n = 70).

Parameters	n	Minimum	Maximum	Mean	SD	T-Test
Age (years)	70	31	79.0	52.5	12.2	**
Total family size (number)	70	3.0	16.0	10.2	7.55	**
Farming experience (years)	70	13.0	55.0	27.4	12.9	**
Total number of livestock	70	0.0	64.0	25.3	14.7	**
Total farmland size (ha)	70	0.61	15.4	3.25	2.42	**
Farmland size owned by HH (ha)	70	0.00	6.27	1.13	1.37	**
Farmland size inherited (ha)	70	0.00	13.4	1.96	2.41	**
Farmland rented (ha)	70	0.00	1.62	0.17	0.40	**
Irrigated land size (ha)	70	0.00	0.00	0.00	0.00	ns
Ave. monthly income from Agriculture (cedis)^a^	70	113	1450	562	82.0	**
Ave. monthly off-farm income (cedis)	70	0.00	1200	398	390	**
Farm distance from main road (km)	70	0.10	7.00	1.60	1.50	**
Main road distance from home (km)	70	0.10	6.00	1.00	1.00	**
Maize yield in bag per acre (tons ha^-^)	70	0.25	5.00	1.50	0.94	**
Maize- NPK Fertilizer rate applied (kg ha^-1^)	70	0.00	250	75	35	**
Maize- Sulphate ammonia rate applied (kg ha^-1^)	70	0.00	250	100	49	*
Maize- Manure/compost rate applied (tons ha^-1^)	70	0.00	1.25	0.750	0.25	ns
Maize- Urea rate applied (kg ha^-1^)	70	0.00	250	100	25	*
Time of NPK application (weeks after planting = WAP)	27	0.00	6.00	3.17	1.71	**
Time of urea application (WAP)	16	0.00	7.00	4.53	2.38	**
Time of sulphate ammonia application (WAP)	10	0.00	7.00	4.90	1.97	**
Time of manure/compost application (WAP)	0	0.00	0.00	0.00	0.00.	Ns

n, number of respondents; SD, standard deviation; manure/compost is applied at 0 WAP which is at planting time.

** and *, significant at P ≤ 0.01 and P ≤ 0.05, respectively; ns, non-significant at P > 0.05.

^a^During the study period, the average conversion rate of 1 US Dollar = 4.00 cedis, which is the currency of Ghana was used.

Source: Own Survey Data (2017).

In the Eastern Region, the farmers reported that maize yield varied significantly between 0.25 and 5 tons ha^-1^, with a mean value of 1.5 tons ha^-1^. The mean and maximum maize yields in the study village of this region are lower than the estimated achievable yield potential of 6.0 tons ha^-1^ which is reported in the conditions of Ghana [6, 60]. According to the respondents of this study, a higher maize yield was reported in the Eastern Region than in the Northern Region of Ghana (Tables 4 and 5), which could be associated with the severity of soil degradation and effects of climate variability. According to the respondents in the study village from the Eastern Region of Ghana, household head average monthly income from agriculture varied between 30 and 1450 Ghana cedis (7.50 to 363 US Dollar) (Table 4). Such significant variation of income from agriculture could be associated with differences in soil and crop management practices such as fertilizer, weeding, besides to the farmland size possessed by the farmers.

**Table 5:** Quantitative results of respondents’ socioeconomic, farm and institutional attributes of the study village from the Northern Region of Ghana (n = 60).

Parameters	n	Minimum	Maximum	Mean	SD	T-Test
Age (years)	60	25.0	65.0	46	9.70	**
Total family size (number)	60	2.00	19.0	8	3.60	**
Farming experience (years)	60	10.0	50.0	29	10.1	**
Total number of livestock	60	0.00	60.0	22	15.7	**
Total farmland size (ha)	60	0.61	14.2	2.97	2.32	**
Farmland size owned by household head (ha)	60	0.00	6.10	0.425	1.21	**
Farmland size inherited (ha)	60	0.00	11.3	2.02	1.88	**
Farmland rented (ha)	60	0.00	14.2	0.55	2.02	*
Irrigated land size (ha)	60	0.00	2.50	0.04	0.32	**
Ave. monthly income from Agriculture (cedis)^a^	60	105	1380	459.0	351	**
Ave. monthly off-farm income (cedis)	60	0.00	1000	398.0	227	**
Farm distance from main road (km)	60	0.50	15.0	3.65	3.64	**
Main road distance from home (km)	60	0.50	6.00	1.27	1.06	**
Maize yield (tons ha^-^)	60	0.50	4.00	1.20	0.29	*
Rice yield (tons ha^-^)	45	0.52	5.50	1.40	1.10	*
Sorghum yield (tons ha^-^)	3	0.29	0.75	0.50	0.18	ns
Groundnut yield (tons ha^-^)	24	0.75	2.50	1.50	0.56	*
Cassava yield (tons ha^-^)	6	0.98	3.70	2.50	1.12	**
Pepper yield (tons ha^-^)	5	0.50	5.60	3.40	1.37	*
Maize- NPK Fertilizer rate applied (kg ha^-1^)	60	0.00	250	225	85	**
Maize- Sulphate ammonia rate applied (kg ha^-1^)	60	0.00	250	85	70	**
Maize-Manure/compost rate applied (tons ha^-^)	60	0.00	10.0	4.0	2.20	ns
Time of NPK use to maize (weeks after planting = WAP)	59	2.00	6.00	3.20	0.56	**
Time of sulphate ammonia application to maize (WAP)	37	5.00	8.00	6.40	0.79	**
Time of manure/compost application to maize (WAP)	39	0.00	0.00	0.00	0.00	ns
Rice- NPK fertilizer rate applied (kg ha^-1^)	42	0.00	3.00	1.55	0.75	**
Rice- Sulphate ammonia rate applied (kg ha^-1^)	40	0.00	3.00	0.59	0.67	**
Rice- Urea rate of applied (kg ha^-1^)	41	0.00	100	17	8.00	**

n, number of respondents; SD, standard deviation; manure/compost is applied at 0 WAP which is at planting time.

** and *, significant at P ≤ 0.01 and P ≤ 0.05, respectively; ns, non-significant at P > 0.05.

^a^During the study period, the average conversion rate of 1 US Dollar = 4.00 cedis, which is the currency of Ghana was used.

Source: Own Survey Data (2017).

The rate of chemical fertilizer (e.g., NPK, sulphate ammonia fertilizers, urea) used by the farmers varied significantly between 0 and 250 kg ha^-1^, with a mean value of 100 kg ha^-1^ in the study village from the Eastern Region of Ghana. The highest rate of fertilizer was reported for NPK and sulphate ammonia fertilizers followed by the urea fertilizer in the study village from the Eastern Region. This variability in fertilizer rates and time of application depend mainly on the financial capacity of the farmers in which rich farmers could be applied more fertilizer than the poor once. In addition, farmers’ awareness on the expected profit to be achieved due to the application of fertilizer could influence significantly the rate and time of fertilizer being applied by a farmer.

The time of fertilizer application by farmers was reported to vary between 4 and 5 weeks after planting time. About 65% of the respondents used manure as soil management practice just at planting time or before the planting time in the study village from the Northern Region whereas all of the respondents in the study village from the Eastern Region of Ghana did not use manure at their farmland (Tables 4 and 5). This study generalized that variation in farmers quantitative attributes such as age and farming experiences could lead to differences in their perception on soil degradation due to erosion in the study village from the Eastern Region of Ghana.

In the study village from the Northern Region of Ghana, age of the respondents varied significantly between 25 and 65 years, total family size from 2 to 19 and farming experiences from 10 to 50 years. The total family size in this region study village was higher than that of the village in the Eastern Region of Ghana. The bigger family size in the study village from the Northern Region is due to the respondents’ Muslim religion which allows for a husband to have more than one wife. A bigger family size could be the reason for having smaller land size in the study village from the Northern Region than in the Eastern Region. The total livestock owned by a household head farmer ranged from 0 to 60 in the Northern Region study village. In this region, the total farmland size varied significantly between 0.61 and 14.2 ha, with a mean value of 2.97 ha. This mean value is nearly similar to the report by Peprah et al. [16] who have reported that more than 70% of the farmers in Ghana could cultivate about 3 ha of land.

In the study village from the Northern Region, there were some farmers who practiced irrigation upto 2.50 ha of land. According to the respondents in this region, the minimum and maximum incomes from agriculture and off-farm activities varied significantly among the respondents (Table 5). According to the respondents (100%), the mean monthly income from agriculture was 459 cedis (115 US Dollar) and from off-farm activities was 398 cedis (99.5 US Dollar). The significant variability in the respondents’ perception on crop grain yields, fertilizer rates and time of fertilizer application in the study village from the Northern Region is also shown in Table 5. According to all the respondents (100%) for maize and 75% of them for rice, the mean yield of the main crop (maize) and rice in the Northern Region were reported as 1.20 and 1.40 tons ha^-1^, respectively. In this region study village, yield of maize and rice ranged from 0.50 to 4.00 tons ha^-1^, and 0.52 to 5.50 tons ha^-1^, respectively. The mean yield of maize reported at farmers field (1.2 tons ha^-1^) is below the national yield (1.7 tons ha^-1^) [60] and is also below the attainable yield potential from research trials reported between 4 to 6.0 tons ha^-1^ for Ghana [6, 60–62]. Farmers confirmed that such lower yield could be associated with the low rate of fertilizer application coupled with climate related effects in Ghana in which this is consistent with the reports reported by previous papers [6, 7, 61, 63].

For maize, the respondents applied sulphate ammonia or NPK fertilizer rates that significantly varied between 0 and 250 kg ha^-1^, which indicates that there are many farmers who did not use fertilizer on their farmlands in the study village from the Northern Region. The majority of the farmers (65%) reported that they used manure as a source of fertilizer even though the rate is lower than 4 ton ha^-1^. In support with the present result of fertilizer variability and its implication on production, Tesfahunegn et al. [64] have reported that a lower crop grain yield could be associated with poor soil and crop management in the Northern Ethiopia conditions. Generally, the implication of this study is that the significant variability in the quantitative attributes among the farmers could contribute towards variation in their perception on soil erosion.

### Farmers’ perception on soil erosion, sedimentation and causes of erosion

In the study village from the Eastern Region of Ghana, significantly higher proportions of the farmers’ (95.7%) perceived that soil erosion is a serious problem. The rest of the respondents’ perceived that erosion is not a problem at their locality. Some of the respondents (24.3%) also perceived that there is sedimentation problem on their field. This study indicates that the proportions of farmers perceived on soil erosion as a problem are higher than those who perceived on sedimentation. The participants in the group discussion also fully agreed that erosion is a serious problem in both study villages as compared to sedimentation. The implication is that priority should be given to develop strategy that reduces the problem of erosion in the study area.

**Fig 2:**
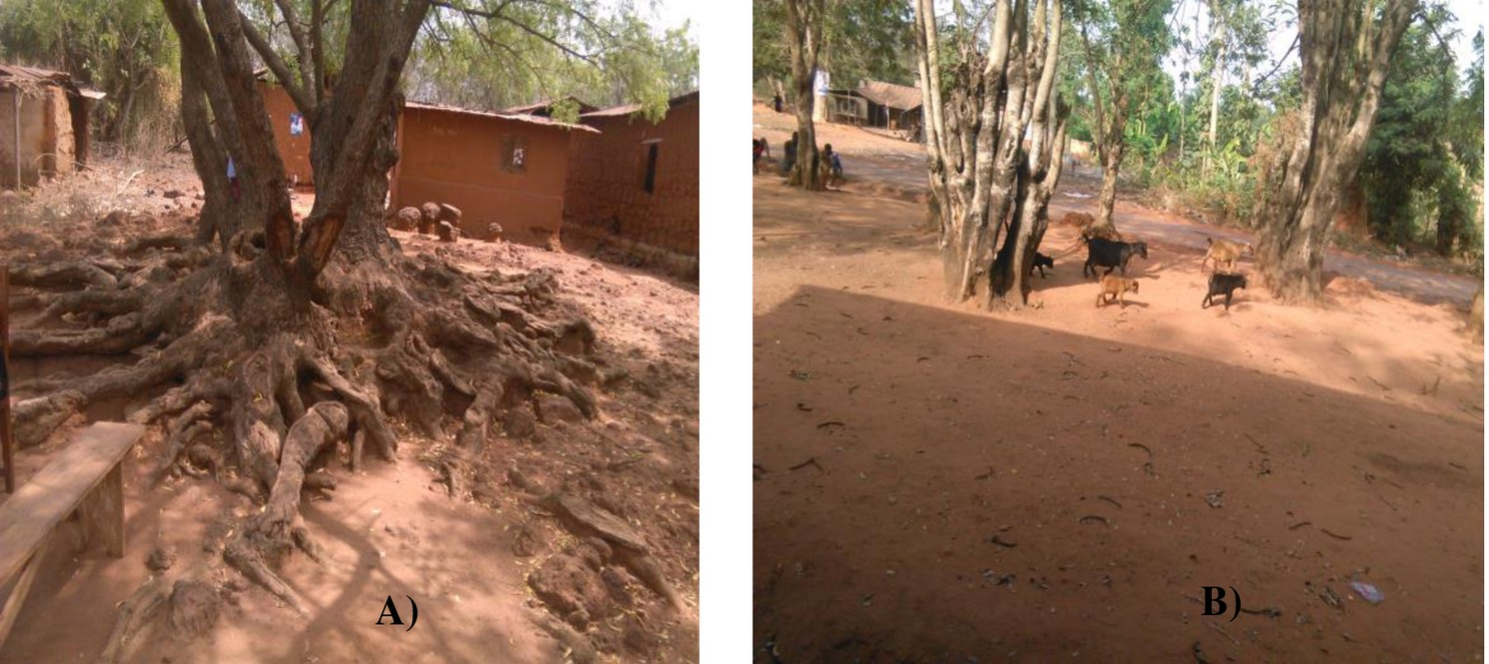
Homestead land with severe erosion indicators in the Eastern Region of Ghana: **A)** Root exposure, and **B)** Loose soil due to overstocking of livestock throughout the year.

According to a significantly higher proportion of the respondents (80%) from the study village in the Easter Region, erosion is perceived as a severe problem at homestead land (Fig 2A), followed by distance arable land (70.0%) as compared to the other land use types (grazing, protected, distance arable land) (Table 6). This was supported by the participants in the group discussion who reported that a severe problem of erosion at homestead land in the study village from the Eastern Region. The reason could be mainly associated with the existence of poor land management practices on gentle to steep slope land coupled with over-stocking of livestock at homestead throughout the year (Fig 2B).

**Table 6:** Farmers’ perception on erosion and sedimentation problems and erosion causes in the selected villages from the Eastern and Northern Regions of Ghana.

Problem	Eastern Region respondents			Northern Region respondents		
	Yes (%)	No (%)	χ2	Yes (%)	No (%)	χ2
Erosion	67 (95.7)	3 (4.3)	**	52 (86.7)	8 (13.3)	**
Sedimentation	17 (24.3)	53 (75.7)	*	22 (36.7)	38 (63.3)	*
**Severe erosion problem**	**Yes (%)**	**No (%)**	χ2	**Yes (%)**	**No (%)**	χ2
Homestead land	56 (80.0)	14 (20.0)	**	2 (3.3)	58 (96.7)	**
Distance arable land	49 (70.0)	21 (30.0)	*	51 (85.0)	9 (15.0)	**
Grazing land	11 (15.3)	59 (84.7)	**	28 (46.7)	32 (53.3)	ns
Protected (closed) land	1 (1.4)	69 (98.6)	**	0 (0.0)	60 (100)	**
**Causes of erosion**	**Yes (%)**	**No (%)**	χ2	**Yes (%)**	**No (%)**	χ2
Improper farming /over- cultivation	39 (55.7)	31 (44.3)	ns	48 (80.0)	12 (20.0)	**
Slope/terrain dissection	46 (65.7)	24 (34.3)	*	28 (46.7)	32 (53.3)	ns
Deforestation	64 (91.4)	6 (8.6)	**	50 (83.3)	10 (16.7)	**
Heavy rainfall	39 (55.7)	31 (44.3)	ns	52 (86.7)	8 (13.3)	**
Absence of SWC practices	40 (57.1)	30 (42.9)	ns	40 (66.7)	20 (33.3)	*
Soil being erodible	19 (27.1)	51 (72.9)	*	6 (10.0)	64 (90.0)	**
Overgrazing	7 (10.0)	63 (90.0)	**	30 (51.7)	30 (48.3)	ns

Values in parentheses are percentages of respondents; χ2, chi-square test

** and *, significant at P ≤ 0.01 and P ≤ 0.05, respectively; ns, non-significant at P > 0.05; SWC, Soil and Water Conservation.

According to the majority of the respondents, over-cultivation (unsuitable farming practices such as conventional tillage, bush burning, and poor soil management), terrain aspects (steep slope), deforestation and heavy rainfall events were perceived as the major causes of severe soil erosion in the study village from the Eastern Region of Ghana (Table 6). Consistent with the present result, past reports have described that deforestation for charcoal and firewood and poor agricultural practices are the main causes for the widespread of degradation due to erosion in many Sub-Saharan Africa countries [38, 65, 66]. In agreement to the present results on the causes of erosion, reports from Ghana have also reported that deforestation (clearing of woodlands and forests) and unsustainable arable farming techniques are reported as the proximate causes of erosion [5, 67]. The same authors have also stated that overgrazing, population growth and poverty are the proximate causes of erosion, but such causes are not identified as the direct causes of erosion by the respondents in the study village from the Eastern Region. Similarly, Tesfahunegn [38] has reported the perception of farmers on poverty as the indirect cause of land degradation in the northern Ethiopia study catchment.

In the study village from the Eastern Region, significantly lower proportions of the farmers were identified that erodible soil condition (fine sand dominated) and overgrazing as the major causes of severe erosion (Table 6). Particularly, overgrazing was not identified by the majority of the farmers as a major cause of erosion in the study village of this region. Such result is contrasted with the reports from Aniah et al. [5]; Nigussie et al. [48] and Bukari et al. [67], who reported that uncontrolled grazing is part of the main causes to result severe erosion. Similarly, according to Nigussie et al. [48], Taddese et al. [68]; and Alemayehu et al. [69], grazing pressure has reported as the main contributor to severe erosion in the highlands of Ethiopia as this practice decreases vegetation cover and increases soil compaction. Such effects lead to low infiltration rate and increases runoff and soil loss. Thus, on the basis of the present study result, awareness creation events for farmers and extension officers should be given on the need to implement SWC practices targeting to erosion prone areas and the consequences of overgrazing towards soil degradation due to erosion in the Eastern Region of Ghana.

In the study village from the Northern Region of Ghana, erosion as a severe problem was perceived by significantly higher proportions of respondents (86.7%) than those who did not perceive erosion. In line with this, all the group discussion participants agreed that soil erosion is a severe problem in this region. Sedimentation was also mentioned as part of the serious problem by significantly lower (36.7%) proportions of the respondents. This indicates that more attention should be given to soil erosion processes than sedimentation in the northern Region. Significantly higher proportions of the respondents reported that the problems of erosion and sedimentation in the Northern Region of Ghana study village were more noticed at distance farmlands than the homestead land.

In the study village from the Northern Region, the major causes of severe erosion perceived by most of the farmers were over-cultivation, deforestation and heavy rainfall within a short period of time. In line with the present finding on the causes of soil erosion in this region, previous reports have shown that anthropogenic factors such as over-cultivation and inappropriate farming practices have reported to lead towards significantly severe degradation in Ethiopia as reported by Tesfahunegn [38], in Sudan by Abdi et al. [70], and in other Sub-Sahara Africa countries by Kiage [24]; Kimaru and Juma [71] and Aksakal et al. [72].

Absence of SWC practices was perceived as a cause for a severe problem of erosion by significantly higher proportions of the respondents as such practices are rarely implemented in the study villages from both regions (Table 6). Few of the farmers who practiced SWC such as ditches and soil bund were learned about such practices from their parents and neighbours. Some respondents (less than 30%) also learned SWC practices from extension staffs in both regions. The main reason why SWC practices have not yet widely practiced could be attributed to lack of technical skill and its labor-intensive demand while labor shortage has become a question of many farmers in developing countries [3, 38, 73].

### Farmers’ perception on erosion severity and indicators of erosion

**Fig 3:**
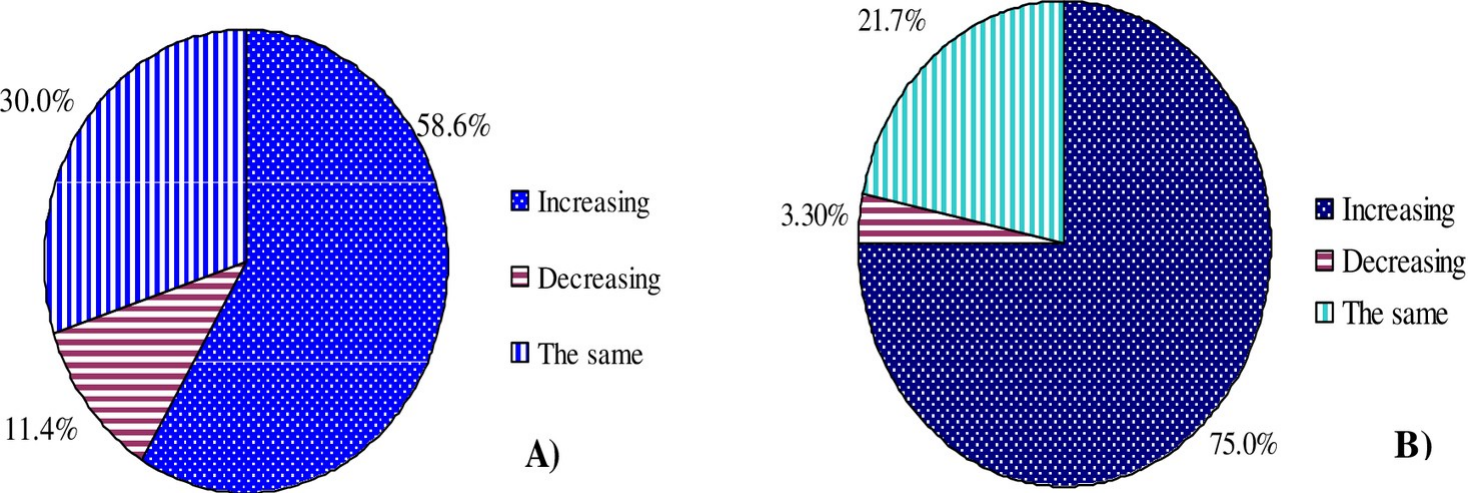
Farmers’ perception on the trend of erosion severity rate (increasing, same, and decreasing) since the past 10 years in the study villages: **(A)** In the Eastern Region, and **(B)** In the Northern Region of Ghana.

In both the study regions, farmers’ perception on the trend of the rate of erosion is shown in Fig 3A and 3B. In the study villages from the Eastern and Northern Regions of Ghana, 58.6 and 75.0% of the farmers perceived, respectively, that soil erosion severity has been increased since the past 10-years. This indicates that the trend on the severity of soil erosion is more perceived by the respondents from the study village in the Northern Region than in the Eastern Region of Ghana. All the participants in group discussion from both regions agreed that soil erosion severity increased from time to time, but the rate of the severity could not be uniform across the years and sites. On the other hand, some respondents from the study village in the Eastern Region (11.4%) and Northern Region of Ghana (3.3%) perceived that the severity of erosion rate has been decreasing from time to time since the past 10 years. Such perception on the decrement of erosion severity by the farmers was described by the continued implementation of some practices, e.g., minimum tillage, crop residues, fertilizer, ditches.

In addition, the chi-square test showed that the proportions of respondents who perceived on some of the indicators of soil degradation due to erosion were significantly higher than those who did not perceive them in the study villages of the two regions (Table 7). In the study village from the Eastern Region, significantly higher proportions of the respondents perceived that the most frequently noted indicators of soil erosion were declined agricultural productivity (98.6%), shallow soil depth (91.4%), presence of rill erosion (87.1%), sheet erosion (85.7%), and change in soil color (77.1%). Similarly, in the Northern Region, significantly higher proportions of the respondents perceived that declined in agricultural productivity (90.0%), shallow soil depth (88.3%), presence of rill erosion (86.7%), soil loss from farm land (86.7%) and change in soil color (85.0%) were the most important indicators of water erosion severity. In this region, the proportions of farmers who perceived sediment deposition, loss of fertilizer and loss of seed planted as indicators of severity of erosion were found significantly higher than those who did not perceive such indicators (Table 7).

**Table 7:** Farmers’ perception on indicators or consequences of soil erosion in the study villages from the Eastern and Northern Regions of Ghana.

Erosion indicator	Eastern Region respondents			Northern Region respondents		
	Yes (%)	No (%)	χ2	Yes (%)	No (%)	χ2
Sheet erosion	60 (85.7)	10 (14.3)	**	39 (65.0)	25 (35.0)	*
Sediment in ditches/ furrows	11 (15.7)	59 (84.3)	**	22 (36.7)	38 (63.3)	*
Rill erosion	61 (87.1)	9 (12.9)	**	52 (86.7)	8 (13.3)	**
Surface pans	31 (44.3)	39 (55.7)	ns	6 (10.0)	54 (90.0)	**
Gullies	34 (48.6)	36 (51.4)	ns	15 (25)	45 (75.0)	*
Pedestals	37 (52.9)	33 (47.1)	ns	10 (16.7)	50 (83.3)	**
Flooding	24 (34.3)	46 (65.7)	*	26 (43.3)	34 (56.7)	ns
Water logging	4 (5.7)	66 (94.3)	**	7 (11.7)	53 (88.3)	**
Shallow soil depth	64 (91.4)	6 (8.6)	**	53 (88.3)	8 (11.7)	**
Declined productivity (yield)	69 (98.6)	1 (1.4)	**	54 (90.0)	6 (10.0)	**
Changes in crop type growing	21 (30.0)	49 (70.0)	*	12 (20.0)	48 (80.0)	**
Reduced farm plot size	26 (37.1)	41 (62.9)	*	36 (60.0)	24 (40.0)	ns
Soil lost from farmland	45 (64.3)	25 (35.7)	*	52 (86.7)	8 (13.3)	**
Sediment deposition	23 (32.9)	47 (67.1)	*	35 (58.3)	25 (41.7)	ns
Loss of fertilizer	16 (22.9)	54 (77.1)	**	39 (65.0)	21 (25.0)	*
Loss of seeds planted	20 (28.6)	50 (71.4)	*	34 (56.7)	26 (43.3)	ns
Soil become coarser or stony	31 (44.3)	39 (55.7)	ns	21 (35.0)	39 (65.0)	*
Change in soil color	54 (77.1)	16 (22.9)	**	51 (85.0)	9 (15.0)	**

Values in parentheses are percentages of respondents; χ2, chi-square test

** and *, significant at P ≤ 0.01 and P ≤ 0.05, respectively; ns, non-significant at P > 0.05.

**Fig 4:**
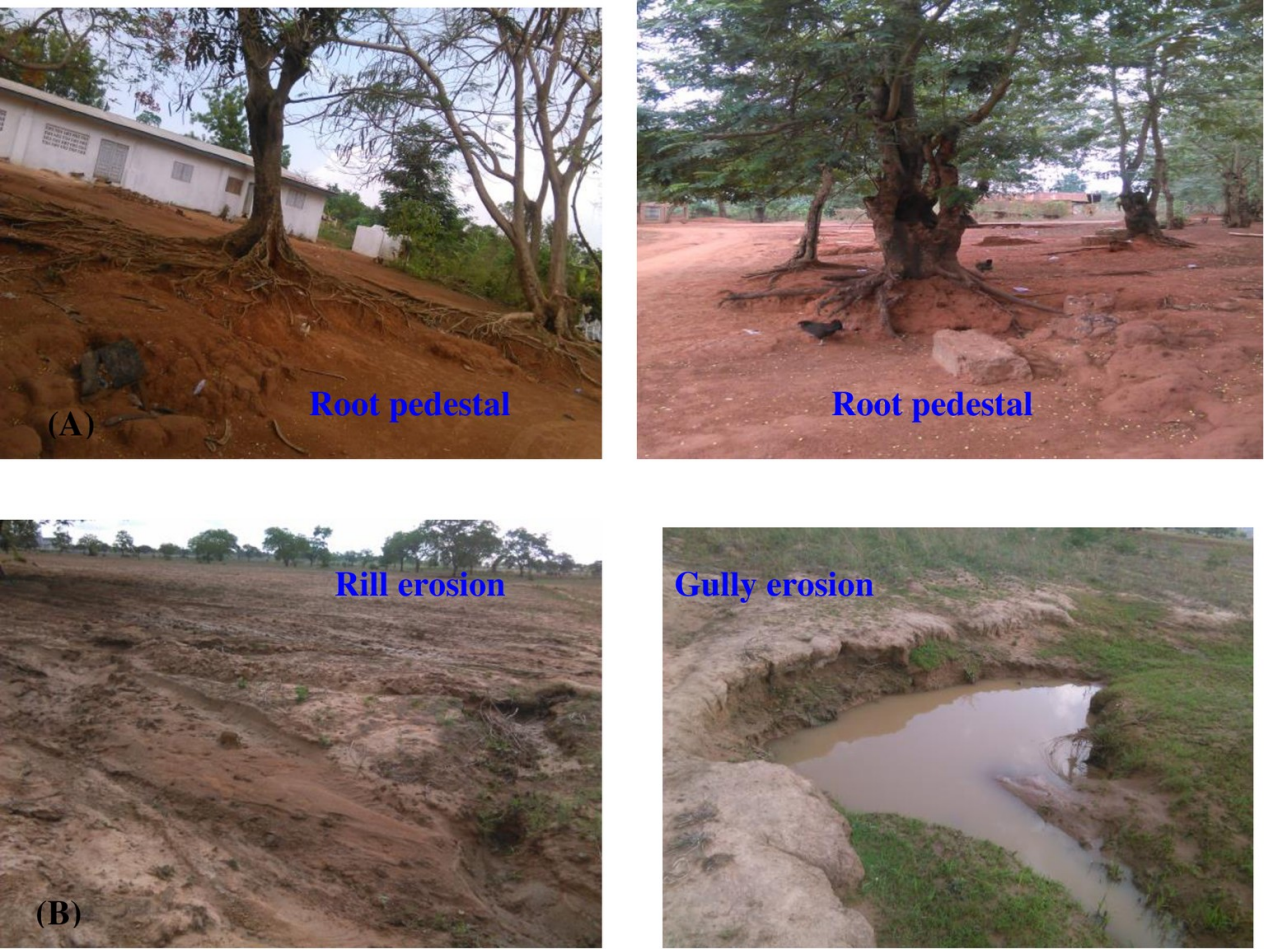
Indicators of erosion severity: (A) Pedastals (exposure of tree roots due to erosion in the Eastern Region), and (B) rill and gully erosion from the Northern Region as. (Source: Gebreyesus Brhane Tesfahunegn, May 2017).

Other erosion indicators such as root pedestals around homesteads and rills and gullies at distance farms were also widely observed at field level in both regions (Fig 4). Consistent with the present erosion indicators, Bukari et al. [67] have reported that farmers perceived sheet erosion (68%) and gully erosion (60%) as the most frequently noted indicators of erosion severity. Several other researchers elsewhere have reported in Africa similar soil degradation (erosion) indicators even though there are some variability on the rank of their importance spatially and temporally (e.g., [74–76]).

**Fig 5:**
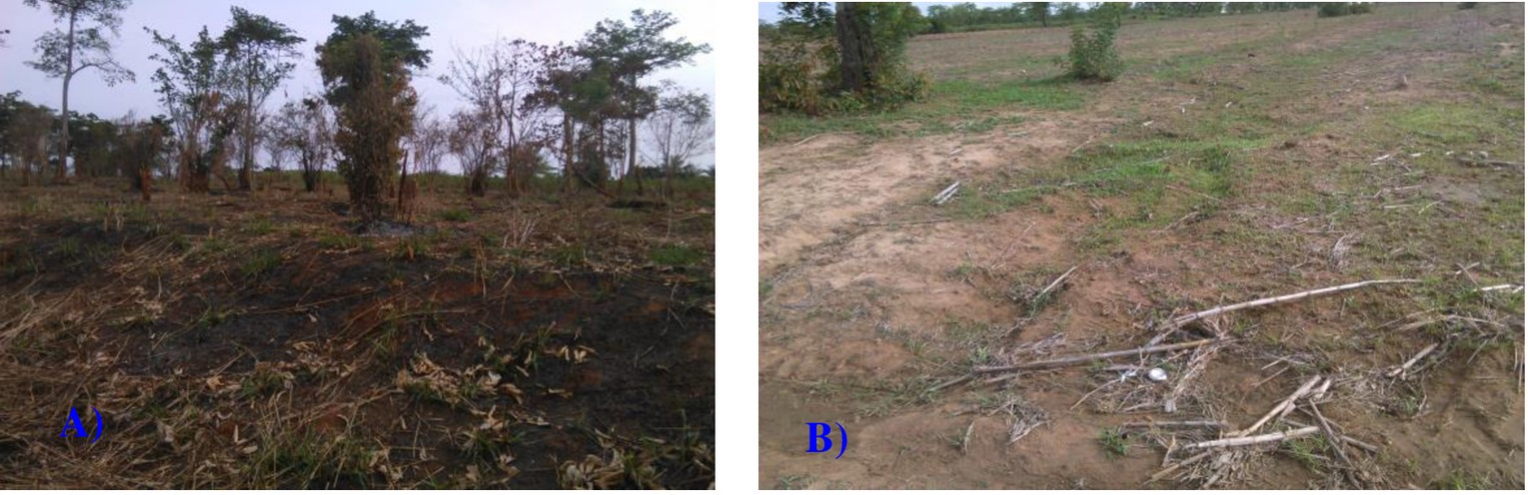
Arable land in the study villages: A) Bush fire with no SWC practice in the Eastern Region, and B) Land without SWC practice in the Northern Region of Ghana. (Source: Gebreyesus Brhane Tesfahunegn, May 2017).

In the study village from the Eastern Region of Ghana, 82.8 and 20% of the respondents replied that farmers were discussed about erosion severity and indicators with their neighbours and extension staffs, respectively. In this region study village, about 81.4% of the respondents replied that farmers were involved to some extent on self-initiated soil and water conservation (SWC) practices in their fields. From the study village in the Northern Region of Ghana perspectives, 86.7% and 18.3% of the respondents were discussed with their neighbours and extension staff, respectively. In this region, significantly higher proportions of the respondents (85%) were attempted to practice SWC practices on their farm lands. However, it was observed that the scope and practical application of SWC practices at field scale by the farmers is too limited in intensity and area coverage (Fig 5). This could be associated with the very weak farmers-extension linkage to introduce SWC practices to wider users in the study villages in Eastern and Northern Regions of Ghana.

### Determinants of farmers’ perception on soil erosion

In the study village from the Eastern Region of Ghana, the binary logistic regression analysis indicated that the most determinant six variables for farmers to perceive soil erosion were age, education, farming experience, total farmland size, farmland slope, and access to extension services (Table 8). The model correct prediction value of the logistic regression analysis of farmers’ perception on soil erosion in this region is explained by 86%. The contribution of the determinant variables, provided that all the other variables remained constant, showed significant relationships with the likelihood of farmers being perceived on soil erosion. Such relationships are explained using the odds ratios of the independent variables, i.e., farming experiences, age, farmland slope, access to extension services, total farmland size, and education as 3.264, 2.825, 2.528, 2.281, 2.162, and 1.916, respectively. The odds ratio of the explanatory variable farmer farming experiences which is 3.264 indicates that all the other variables held constant for every one-unit increase in this independent variable, the likelihood of a farmer for being perceived on soil degradation due erosion increases by 3.264 folds. In a similar way, the odds ratios of the other determinant variables described on the above can be interpreted.

**Table 8:** Binary logistic analysis of determinant variables for farmers’ perception on soil erosion as dependent variable in the study villages from the Eastern and Northern Regions of Ghana.

Variables	Eastern Region (n = 70)			Northern Region (n = 609		
	β	Exp(B)^a^	Sig.	β	Exp(B)^a^	Sig.
Gender	0.67	0.190	0.295	0.49	0.580	0.472
Age	2.48	2.825	0.002**	2.53	2.387	0.020*
Marital status	0.93	0.176	0.237	0.73	0.689	0.365
Education status	1.71	1.961	0.031*	2.94	2.950	0.002**
Total family size	1.20	1.008	0.210	1.83	2.411	0.018*
Farming experience	2.70	3.264	0.001**	3.15	3.195	0.001**
Total livestock owned	0.26	0.774	0.462	0.58	1.186	0.184
Total farmland size	2.05	2.162	0.020*	2.40	2.570	0.016*
Farmland slope	2.43	2.528	0.003**	1.49	1.200	0.173
Farmland ownership	1.33	0.013	0.450	1.27	1.449	0.116
Land security/tenure	0.69	0.968	0.360	1.53	1.162	0.192
Off-farm activity	-1.70	1.685	0.120	-2.12	2.353	0.020*
Agricultural income	1.01	1.008	0.570	1.61	1.357	0.156
Farm distance from main road	-1.00	0.814	0.560	-1.13	1.271	0.170
Farm distance from home	-1.20	0.110	0.590	-0.66	0.705	0.395
Access to extension services	1.59	2.281	0.018*	0.34	0.600	0.512
Access to credit	0.86	0.590	0.620	1.07	0.764	0.283
Access to radio	0.73	0.480	0.631	2.864	2.360	0.020*
Membership to association	1.04	0.561	0.350	0.63	0.533	0.354
Constant	4.232	69.00	0.001	3.37	29.0	0.001
Model chi-square (*χ*^*2*^)	87		0.001	85.0		0.001
Model Nagelkerke R^2^	0.89		0.001	0.88		0.001
Model correct prediction	86%		0.001	84%		0.001

β, Estimated coefficient; R^2^, Coefficient of determination.

** Significant at probability level, p ≤ 0·01. * Significant at p ≤ 0·05; Values without asterisks are non-significant at p>0·05.

^a^ Exp(β) is the ratio of change in the odds of the event of interest to a one-unit change in the predictor [77, 78].

Source: Own Field Survey Data (2017).

In line with this finding from the study village in the Eastern Region, previous reports have reported that slope is found to have a positive and significant effect on soil erosion severity, in which this indicates that farmers having farmland on gentle to steep slope areas are more likely to perceive the impact of slope on the severity of soil erosion [48, 79]. Consistent with this study on the significant effect of farmers’ access to extension services on soil erosion perception, existing reports (e.g., [48, 77] have shown that this variable could influence positively and significantly farmers perception on soil erosion. This could be attributed to the fact that access to extension services such as training, knowledge and technology sharing opportunities can enhance farmers’ perception to look for options of soil and water conservation and management practices against erosion.

In the study village from the Northern Region of Ghana, the binary logistic model correct prediction of the relationship between the dependent and the determinant independent variables is explained by 84%. In this region, the most important variables that significantly influenced farmers’ perception on soil erosion were age, education, total family size, farming experience, total farm size, off-farm activity, and access to radio (Table 8). Farmers who involved the majority of their time on off-farm activities showed a significantly lower perception on soil degradation due to erosion. On the other hand, the remaining determinant explanatory variables increased significantly the farmers’ perception on soil erosion. Such significant relationships between the determinant variables and the dependent variable were explained using the odds ratios of 3.195, 2.950, 2.570, 2.411, 2.387, 2.353, and 2.360 for farming experiences, education, total farm size, total family size, age, access to radio, and off-farm activities, respectively.

All the other variables kept constant for every one-unit increased in the off-farm activity (independent variable), the likelihood of a farmer being perceived on soil degradation due to erosion decreases by 2.360 folds. Similarly, provided that all the other variables held constant for every one-unit increased in the farming experience (independent variable), the likelihood of a farmer being perceived on soil erosion increases by 3.195 folds. The implication of the odds ratios of the other determinant variable can be interpreted using this example. Access to radio was found to affect positively and significantly farmers perception on soil erosion in which this variable is likely enables farmers’ to get knowledge and information about technology related to land management options that tackle to erosion [48, 80, 81].

In both study villages from the two regions, the common determinant variables that significantly influenced the farmers’ perception on soil erosion were age, education, farming experiences and farmland size (Table 8). In agreement to the present study, education status and farmland size have reported to be influenced significantly farmers perception on soil erosion in the Upper Blue Nile Basin (Ethiopia) [48, 82, 83]. Because education can enhance farmers’ ability to process new information about causes and indicators of erosion and the possible management practices. In agreement to this study result from the two regions of Ghana, others have reported that factors such as farmers’ farming experiences on soil-and-crop management, water harvesting, soil conservation practices, could contribute more likely to have farmers positive and significant perception on soil erosion [38, 42, 48, 84]. Consistent with the present result of the determinant variable farmland size that positively influences on farmers perception on soil erosion, the larger the cultivated field, the higher is the likelihood of farmers to observe sheet erosion, rills, surface runoff, sediment deposition and redeposition [48, 85].

According to Nigussie et al. [48], the numbers of livestock owned by household head farmers and farmland distance to residence area have reported to influence significantly farmers’ perception on soil erosion. However, such report is contradicted with the present result which reported that the two variables did not influence significantly farmers’ perception on erosion in the two regions study villages. The fact is that more distant farmland received less frequency to be visited by farmer as compared to nearby farmlands in which this can affect negatively farmers’ perception on soil erosion [38, 48]. Generally, in both regions of this study, it has noted that the relationships between many explanatory variables and farmers’ perception on soil erosion (dependent variable) showed non-significant differences (e.g., gender, marital status, land tenured, agricultural income, membership to association). Hence, attention should be given to the determinant variables which influenced significantly farmers’ perception on soil erosion while developing promising landscape management strategy for the study villages’ conditions.

### Implication for developing soil management strategy

This study showed that farmers’ perception on soil degradation due to erosion is a function of the socioeconomic, biophysical, farm and institutional factors. However, the implication of this study is that all such factors are not equally important to influence farmers’ perception on soil erosion in a given village and across the study villages. Thus, attention should be given to the determinant variables in each village while developing site-specific appropriate soil management strategy. The soil management strategy could vary by land use type such as cultivation, grazing, and marginal land and slope differences, besides to the consideration for uncertainty in climate change and variability [86, 87]. Different management strategy that considers the landscape heterogeneity is suggested to be developed. For instance, cultivated land may need soil and water conservation practices such as soil bund alone or integrated with grass and also improved seed with organic and inorganic fertilizers. Similarly, grazing land can demand soil and water conservation structures such as bunds and terracing integrated with forage development activities. On marginal land, management strategy related to deep trench and bench terracing integrated with plantations could be effective [38]. Generally, soil management interventions which are described on the above can be effective when farmers perceived soil degradation and challenges for soil management strategy implementation. To do so, it should be to consider farmers involvement in all decision making processes related to soil management strategy development and its implementation in the study villages’ conditions.

The frequency of contacts that farmers have with agricultural extension agents and peer to pear learning at their locality at field days about erosion problems and the corresponding controlling measures could be improved farmers perception on soil erosion. This is likely true because a greater contact with extension agents could enable farmers’ access to new technology and knowledge-intensive information and develop additional skills related to the implementation of sustainable land management options that reduce erosion drastically. Improving education status of farmers more enhance farmers perception not only towards erosion but also it is crucial to adopt new and modified techniques that reduce soil erosion and thereby improve land productivity [42, 67, 86, 87].

## Conclusion

This study revealed that there were significant differences among the majority of the farmers in socioeconomic, farm and institutional factors in which such variations could influence their perception on soil erosion in the study villages from the Eastern and Northern Regions of Ghana. Some of the factors such as off-farm activity, access to extension services, varied significantly among the respondents in the study village from the Eastern Region but not in the Northern Region of Ghana. Similarly, farmland slope and membership to local association showed significant differences among the respondents in the study village from the Northern Region where as such factors varied non-significantly among the farmers in the Eastern Region. The implication is that variation of such factors among the farmers varied not only within a village but also across the study villages in which this can lead to farmers’ differences of perception on soil erosion. Consequently, significantly higher proportions of the farmers perceived that soil erosion is a serious problem in both study villages in Ghana, but the proportions varied across the two regions which was 95.7% in the Eastern Region and 86.7% in the Northern Region. The proportions of farmers perceived on soil erosion as a problem are higher than those who perceived on sedimentation in both regions, indicating that management strategy is suggested to target on reducing soil erosion.

Significantly higher proportions of the respondents (80%) perceived soil erosion as a severe problem at homestead land, followed by distance arable land (70.0%) in the study village in the Eastern Region of Ghana. On the contrary, significantly higher proportions of the respondents reported that severe erosion and sedimentation in the study village from the Northern Region of Ghana were more noticed at distance farmlands than the homestead land types. This study indicated from both study regions, the majority of the farmers perceived that soil erosion severity has been increased since the past 10-years. The major causes of severe erosion perceived by the majority of the farmers are over-cultivation with poor soil management practices, deforestation and heavy rainfall events in a short period of time.

The most frequently perceived indicators of soil erosion in the Eastern Region study village by significantly higher proportions of the respondents in descending order were declined in agricultural productivity, shallow soil depth, presence of rills, sheet erosion, and change in soil color. Similarly, significantly higher proportions of the respondents perceived that declined productivity or yield, shallow soil depth, presence of rills, soil loss from farm land and change in soil color were perceived as the most important indicators of erosion severity in the study village from the Northern Region. In both regions, the majority of the respondents indicated that they were discussed more frequently about erosion severity and indicators with their neighbours than with extension staffs, implying that there is a need to improve such communication gaps between the farmers and extension workers.

In both of the study villages in both the regions, the common determinant variables that significantly influenced the farmers’ perception on soil erosion were age, education, farming experiences and farmland size. In the study village from the Eastern Region, the other determinant variables were land slope and access to extension services; whereas family size, off-farm activity and access to radio were the additional determinant variables for farmers’ perception on soil erosion in the study village from the Northern Region. Farmers’ perception on erosion causes, severity, trend and indicators should be considered for the development of management actions against erosion. Besides to this, the determinant variables for farmers perception on soil erosion identified in each of the study villages in the two regions should be considered while developing site-specific appropriate erosion management strategy. The management strategy could include integrated approaches (agronomic, biological and physical soil and water conservation practices) and awareness creation forums and activities on local community and extension workers on how to participate and manage erosion sustainably.
